# Total neoadjuvant treatment for MRI-stratified high-risk rectal cancer: a single-center, single-arm, prospective Phase II trial (PKUCH-R02)

**DOI:** 10.1093/gastro/goad017

**Published:** 2023-04-18

**Authors:** Peng-Ju Chen, Lin Wang, Ting-Ting Sun, Yun-Feng Yao, Yi-Fan Peng, Jun Zhao, Tian-Cheng Zhan, Jia–Hua Leng, Yong Cai, Yong-Heng Li, Xiao-Yan Zhang, Ying-Shi Sun, Zhong-Wu Li, Wei-Hu Wang, Ai-Wen Wu

**Affiliations:** Key Laboratory of Carcinogenesis and Translational Research (Ministry of Education), Department of Gastrointestinal Cancer Unit III, Peking University Cancer Hospital & Institute, Beijing, P. R. China; Key Laboratory of Carcinogenesis and Translational Research (Ministry of Education), Department of Gastrointestinal Cancer Unit III, Peking University Cancer Hospital & Institute, Beijing, P. R. China; Key Laboratory of Carcinogenesis and Translational Research (Ministry of Education), Department of Gastrointestinal Cancer Unit III, Peking University Cancer Hospital & Institute, Beijing, P. R. China; Key Laboratory of Carcinogenesis and Translational Research (Ministry of Education), Department of Gastrointestinal Cancer Unit III, Peking University Cancer Hospital & Institute, Beijing, P. R. China; Key Laboratory of Carcinogenesis and Translational Research (Ministry of Education), Department of Gastrointestinal Cancer Unit III, Peking University Cancer Hospital & Institute, Beijing, P. R. China; Key Laboratory of Carcinogenesis and Translational Research (Ministry of Education), Department of Gastrointestinal Cancer Unit III, Peking University Cancer Hospital & Institute, Beijing, P. R. China; Key Laboratory of Carcinogenesis and Translational Research (Ministry of Education), Department of Gastrointestinal Cancer Unit III, Peking University Cancer Hospital & Institute, Beijing, P. R. China; Key Laboratory of Carcinogenesis and Translational Research (Ministry of Education), Department of Gastrointestinal Cancer Unit III, Peking University Cancer Hospital & Institute, Beijing, P. R. China; Key Laboratory of Carcinogenesis and Translational Research (Ministry of Education), Department of Radiation Oncology, Peking University Cancer Hospital & Institute, Beijing, P. R. China; Key Laboratory of Carcinogenesis and Translational Research (Ministry of Education), Department of Radiation Oncology, Peking University Cancer Hospital & Institute, Beijing, P. R. China; Key Laboratory of Carcinogenesis and Translational Research (Ministry of Education), Department of Radiology, Peking University Cancer Hospital & Institute, Beijing, P. R. China; Key Laboratory of Carcinogenesis and Translational Research (Ministry of Education), Department of Radiology, Peking University Cancer Hospital & Institute, Beijing, P. R. China; Key Laboratory of Carcinogenesis and Translational Research (Ministry of Education), Department of Pathology, Peking University Cancer Hospital & Institute, Beijing, P. R. China; Key Laboratory of Carcinogenesis and Translational Research (Ministry of Education), Department of Radiation Oncology, Peking University Cancer Hospital & Institute, Beijing, P. R. China; Key Laboratory of Carcinogenesis and Translational Research (Ministry of Education), Department of Gastrointestinal Cancer Unit III, Peking University Cancer Hospital & Institute, Beijing, P. R. China

**Keywords:** rectal cancer, neoadjuvant chemoradiotherapy, MRI, prognosis, pathological complete response

## Abstract

**Background:**

Induction chemotherapy combined with neoadjuvant chemoradiotherapy has been recommended for patients with high-risk, locally advanced rectal cancer. However, the benefit of more intensive total neoadjuvant treatment (TNT) is unknown. This study aimed to assess the safety and efficacy of induction chemotherapy combined with chemoradiotherapy and consolidation chemotherapy for magnetic resonance imaging-stratified high-risk rectal cancer.

**Methods:**

This was a single-center, single-arm, prospective Phase II trial in Peking University Cancer Hospital (Beijing, China). Patients received three cycles of induction oxaliplatin and capecitabine (CapeOX) followed by chemoradiotherapy and two cycles of consolidation CapeOX. The primary end point was adverse event rate and the second primary end points were 3-year disease-free survival rate, completion of TNT, and pathological downstaging rate.

**Results:**

Between August 2017 and August 2018, 68 rectal cancer patients with at least one high risk factor (cT3c/3d/T4a/T4b, cN2, mesorectal fascia involvement, or extramural venous invasion involvement) were enrolled. The overall compliance of receiving the entire treatment was 88.2% (60/68). All 68 patients received induction chemotherapy, 65 received chemoradiotherapy, and 61 received consolidation chemotherapy. The Grade 3–4 adverse event rate was 30.8% (21/68). Nine patients achieved clinical complete response and then watch and wait. Five patients (7.4%) developed distant metastasis during TNT and received palliative chemotherapy. Fifty patients underwent surgical resection. The complete response rate was 27.9%. After a median follow-up of 49.2 months, the overall 3-year disease-free survival rate was 69.7%.

**Conclusions:**

For patients with high-risk rectal cancer, this TNT regimen can achieve favorable survival and complete response rates but with high toxicity. However, it is necessary to pay attention to the possibility of distant metastasis during the long treatment period.

## Introduction

Neoadjuvant chemoradiotherapy (CRT) followed by total mesorectal excision (TME) and post-operative chemotherapy has been widely accepted as the standard treatment for locally advanced rectal cancer [1–3]. However, long-term follow-up of patients receiving this treatment indicated that CRT provided significant local control but failed to improve disease-free survival (DFS) or overall survival (OS). In recent years, many efforts have been made to reduce distant metastasis, which is considered the major cause of death.

Recent strategies for locally advanced rectal cancer have focused on providing preoperative rather than post-operative chemotherapy, also known as total neoadjuvant treatment (TNT) [4]. The advantages of TNT include increased pathological complete response (pCR), increased tolerance and completion rate of systemic chemotherapy, and improved DFS [5]. So far, several TNT schedules have been proposed, including short- or long-course CRT and consolidation chemotherapy, induction chemotherapy followed by short- or long-course CRT, and CRT followed by consolidation chemotherapy [6]. Although TNT has achieved good results in terms of disease control, its wide application may be limited by a high rate of adverse events. In a recent meta-analysis of TNT, the rate of Grade 3–4 adverse events was 20%–30% [4]. Therefore, the administration of TNT in patients with rectal cancer should be based on risk stratification by magnetic resonance imaging (MRI). Patients with mesorectal fascia-involved (MRF^+^), extramural venous invasion (EMVI^+^), cN2, or cT3c/T3d/T4a/T4b disease are considered to have a high risk of distant metastasis and need more intensive treatment [7–10]. The future trend is that patients with Stage II and Stage III locally advanced rectal cancer may need TNT, but treatment strategies for patients with different risk factors still need to be improved. Therefore, we designed a Phase II, single-center, non-randomized clinical prospective study (ClinicalTrials.gov: NCT02864849) to explore the tolerance and efficacy of induction chemotherapy, intensity-modulated radiotherapy (IMRT)-based CRT, and consolidation chemotherapy for patients with MRI-stratified, locally high-risk rectal cancer.

## Patients and methods

### Study design

We designed this single-center, single-arm, prospective Phase II trial to assess the safety and efficacy of TNT in rectal cancer patients with high risk factors. Clinical evaluation was performed using high-resolution MRI combined with chest CT, abdominal contrast-enhanced CT, and full colonoscopy. High risk factors were defined as cT3c/3d/T4a/T4b, cN2, MRF^+^, and EMVI^+^ disease.

The inclusion criteria were as follows: (i) age 18–75 years; (ii) at least one high risk factor as defined above; (iii) Eastern Collaborative Oncology Group performance status score of 0–1; (iv) rectal adenocarcinoma confirmed by pathology; (v) tumor located <15 cm from the anal verge; (vi) no evidence of distant metastasis; (vii) no history of pelvic radiotherapy or systematic chemotherapy; and (viii) normal bone marrow/liver/kidney function. The following exclusion criteria were used: (i) recurrent rectal cancer; (ii) history of other malignancies; and (iii) pregnancy.

### Study protocol

**Figure 1.**
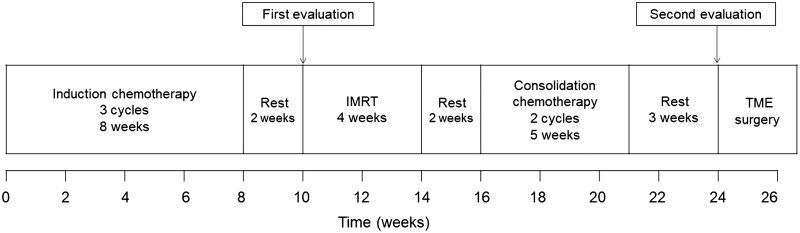
Protocol of the total neoadjuvant treatment. IMRT, intensity-modulated radiation therapy; TME, total mesorectum excision.

This study was approved by the Ethics Committee of the Peking University Cancer Hospital (Beijing, China) and performed in accordance with the Helsinki Declaration (G2015-0816). Written informed consent was obtained from all the patients. The treatment schedule was designed as three cycles of induction chemotherapy, IMRT-based neoadjuvant CRT, two cycles of consolidation chemotherapy, and TME surgery (Figure 1). Induction and consolidation chemotherapy consisted of oxaliplatin 130 mg/m^2^ on Day 1 and capecitabine 1,000 mg/m^2^ twice daily on Days 1–14 in a 3-week cycle. Neoadjuvant CRT consisted of IMRT administered once a day at 2.3 Gy/fraction, 5 days per week, for a total dose of 50.6 Gy in 22 fractions and capecitabine 825 mg/m^2^ twice a day while radiotherapy was ongoing. The detailed procedure for IMRT for rectal cancer has been reported previously [11].

Patients were evaluated twice during TNT, including rectal MRI, chest and abdominal CT, and carcinoembryonic antigen (CEA) assessment. The first evaluation was conducted between the completion of induction chemotherapy and the start of CRT, and the second evaluation was conducted after the completion of treatment. Treatment of patients with distant metastasis was determined after multidisciplinary team discussion. All the patients were recommended for TME after the completion of all treatment for 3 weeks. Patients who were evaluated as having achieved clinical complete response (cCR) and who refused surgery entered a “wait and watch” cohort after providing informed consent. The assessment of cCR was based on digital rectal examination, endoscopy, pelvic MRI, chest and abdominal CT, and CEA assessment [12, 13]. The evaluation of cCR on digital rectal examination was defined as no palpable tumor in patients in whom the tumor was initially palpable. Endoscopic findings of cCR included a flat white scar or nodularity and lack of ulceration with or without telangiectasias. The pelvic MRI of cCR was defined as no residual tumor signal or only fibrosis without any suspicious lymph nodes. For chest CT and abdominal CT, cCR was defined as no evidence of distant metastasis, and a normal CEA was required.

Post-operative complications were defined as any surgical and non-surgical complications occurring within 30 days after surgery based on the Clavien–Dindo classification [14]. Surgical specimens were evaluated by experienced pathologists and the tumor regression grade (TRG) classification was based on the National Comprehensive Cancer Network (NCCN) standard as follows: 0, complete response with no detectable cancer cells; 1, major response with few residual cancer cells; 2, partial response; and 3, no or very little response [15]. The pCR was defined as no residual cancer cells at the primary tumor site or lymph nodes in the pathological specimens after radical surgery. Adjuvant chemotherapy was not performed after radical surgery.

### Follow-up

All patients underwent follow-up, including physical examination, blood testing, and serum CEA assessment, every 3 months for the first 2 years after surgery, then every 6 months for the next 3 years, and once a year thereafter. Colonoscopy was recommended 1, 3, and 5 years after surgery. Chest, abdominal, and pelvic CT were recommended every 3–6 months.

### End points

The primary end point was adverse events according to the Common Terminology Criteria for Adverse Events version 4.0. The secondary end points were 3-year DFS rate, pathological downstaging rate, and compliance of TNT [16]. DFS was defined as the time from treatment until the first cancer-related event or death from any cause.

### Statistical analysis

In the GCR-3 study, the rate of severe adverse events from induction chemotherapy was 42% (radiotherapy 23% and induction chemotherapy 19%) [4]. We expected that the rate of total Grade 3–4 adverse events from chemoradiotherapy and induction plus consolidation chemotherapy in this study would be <25% (IMRT 5% and chemotherapy 20%) based on the results of previous studies [11]. Therefore, a sample size of 67 patients was planned with a two-sided alpha of 0.05 and power of 80%. Taking into account a dropout rate of 10%, ≥75 patients would be included in the study. Patients’ baseline characteristics, adverse event data, and clinicopathological parameters were summarized using descriptive statistics. Statistical analysis was conducted using PASW Statistics (SPSS, IBM Corp., Chicago, IL, USA).

## Results

### Patient characteristics

Between August 2017 and August 2018, 72 MRI-stratified patients with high-risk rectal cancer (cT3c/3d/T4a/T4b, cN2, MRF^+^, or EMVI^+^ disease) were enrolled in this study (Figure 1). Four patients withdrew from the treatment; therefore, 68 patients were eligible for the final analysis. The patients’ characteristics are shown in Table 1. The median age was 57 years. All the patients enrolled in this study had at least one high risk factor. There were 17 (25.0%), 24 (35.3%), and 12 (17.6%) patients who had two, three, and four high risk factors, respectively.

**Table 1.** Characteristic of 68 patients with rectal cancer enrolled in total neoadjuvant treatment

Characteristic	Value
Age, years, median (range)	57 (25–73)
Male, *n* (%)	48 (70.6)
Location (distance for the AV, cm), *n* (%)	
0–5	48 (70.6)
5.1–10	19 (27.9)
10.1–15	1 (1.5)
cT stage, *n* (%)	
T3c	14 (20.6)
T3d	7 (10.3)
T4a	22 (32.4)
T4b	25 (36.8)
cN stage, *n* (%)	
N0	3 (4.4)
N1	11 (6.2)
N2	54 (79.4)
EMVI, *n* (%)	
Positive	25 (36.8)
Negative	43 (63.2)
MRF, *n* (%)	
Positive	40 (58.8)
Negative	28 (41.2)
CEA, *n* (%)	
Elevated	32 (47.1)
Normal	36 (52.9)
High risk factors, *n* (%)	
1	15 (22.1)
2	17 (25)
3	24 (35.3)
4	12 (17.6)

AV, anal verge; EMVI, extramural venous invasion; MRF, mesorectal fasci; CEA, carcinoembryonic antigen.

### Compliance of treatment

In total, 88.2% (60/68) patients completed TNT (including three cycles of induction chemotherapy, 50.6 Gy IMRT, and two cycles of consolidation chemotherapy). All 68 patients received induction chemotherapy, 65 received IMRT, and 61 received consolidation chemotherapy (Table 2). Three patients (4.4%) developed Grade 4 diarrhea after three cycles of induction chemotherapy and then underwent TME without CRT. One patient (1.5%) developed Grade 3 thrombocytopenia after the first cycle of induction chemotherapy and then underwent IMRT and TME. One patient (1.5%) experienced Grade 3 thrombocytopenia after the second cycle of induction chemotherapy and then underwent IMRT and TME. Two (2.9%) patients developed Grade 3 thrombocytopenia and leukopenia after three cycles of induction chemotherapy and then underwent IMRT and TME. One patient (1.5%) experienced Grade 3 thrombocytopenia after one cycle of consolidation chemotherapy and then underwent TME.

**Table 2.** Completion of total neoadjuvant treatment in 68 patients with rectal cancer

Treatment	No. of patients (%)
1*cycle CapeOX+IMRT	1 (1.5)
2*cycles CapeOX+IMRT	1 (1.5)
3*cycles CapeOX	3 (4.4)
3*cycles CapeOX+IMRT	2 (2.9)
3*cycles CapeOX+IMRT+1*cycle CapeOX	1 (1.5)
3*cycles CapeOX+IMRT + 2*cycles CapeOX	60 (88.2)

CapeOX, capecitabine and oxaliplatin; IMRT, intensity-modulated radiotherapy.

**Figure 2.**
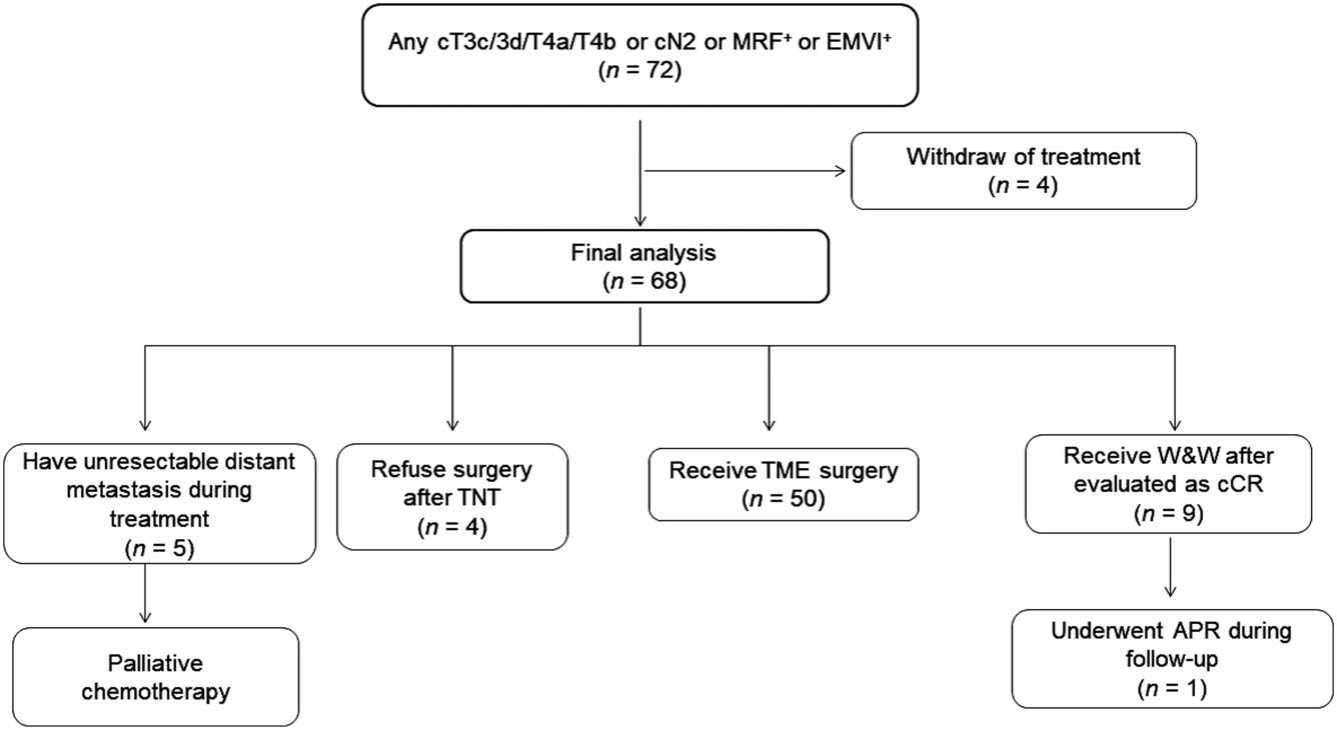
Flow chart of the 72 patients enrolled in the study. MRF, mesorectal fascia; EMVI, extramural venous invasion; TNT, total neoadjuvant treatment; W&W, watch and wait; cCR, clinical complete response; TME, total mesorectal excision; APR, abdominoperineal resection.

**Figure 3.**
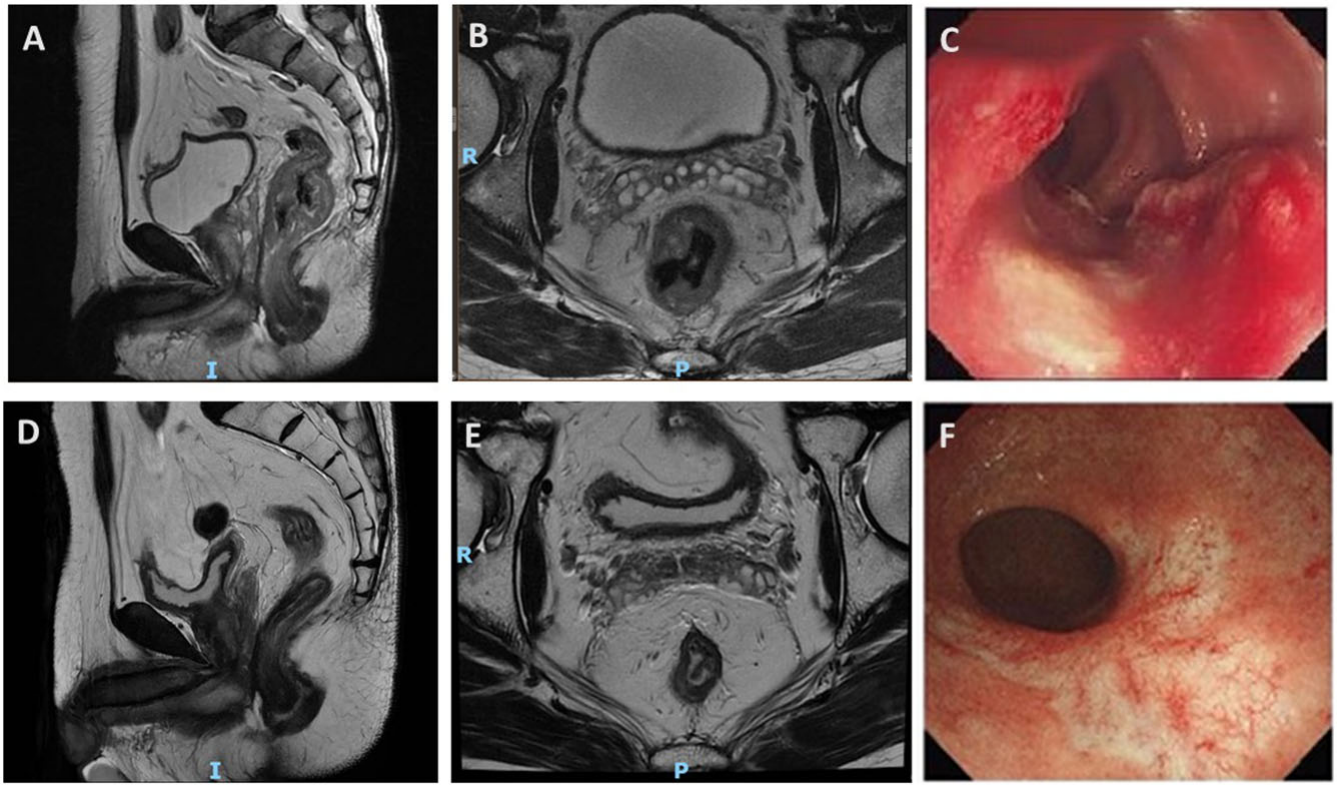
Images from a patient in the watch and wait group. (A) and (B) Sagittal and axial T2-weighted image of the primary tumor before treatment. (C) Endoscopic tumor before treatment. (D) and (E) Sagittal and axial T2-weighted image after TNT. (F) Residual white scar and telangiectasia after TNT at endoscopy. TNT, total neoadjuvant treatment.

The treatment workflow of 68 patients is shown in Figure 2. After the second evaluation, four patients who had residual tumors refused surgery. Nine patients were evaluated as having achieved cCR and entered the “watch and wait” cohort (Figure 3). Fifty patients underwent TME. Three patients developed new distant metastasis after the first evaluation and two patients developed new distant metastasis after the second evaluation (Table 3); these five patients received salvage treatment after multidisciplinary team discussion.

**Table 3.** Baseline clinical features of five patients who developed distant metastasis during total neoadjuvant treatment

Characteristic	Patients having distant metastasis after first evaluation			Patients having distant metastasis after second evaluation	
	No. 1	No. 2	No. 3	No. 4	No. 5
cT stage	cT3c	cT4b	cT3c	cT4a	cT3c
cN stage	cN2	cN1	cN2	cN2	cN2
MRF	+	+	+	–	+
EMVI	–	–	–	–	+
Baseline CEA level (ng/mL)	59.1	94.2	2.9	61.9	19.3
Site of metastasis	Lung	Liver	Lung	Liver	Liver

MRF, mesorectal fascia; EMVI, extramural venous invasion; CEA, carcinoembryonic antigen.

### Adverse events

In total, 65 patients received IMRT and 68 patients received chemotherapy (including induction and consolidation chemotherapy). Grade 3–4 adverse events occurred in 21 (30.8%) patients. Tables 4 and 5 show the details of adverse events from IMRT and chemotherapy. Fifty-six of 65 (86.2%) patients had adverse reactions to IMRT, but most adverse events were Grade 1–2. There was only one patient with a Grade 3 adverse event; no Grade 4 adverse event related to IMRT was observed. The most common acute adverse events associated with IMRT were leukopenia (49.2%) and radiation dermatitis (32.3%). All 68 patients who received chemotherapy developed adverse events and 20 (29.4%) had Grade 3–4 adverse events. The most common acute adverse events from chemotherapy were neurotoxicity (88.2%), fatigue (83.8%), and leukopenia (58.8%).

**Table 4.** Adverse events in total neoadjuvant treatment

Characteristic	Chemoradiotherapy (*n *=* *65)	Chemotherapy in total (*n *=* *68)
Diarrhea	7 (10.8)	18 (26.5)
Vomiting	8 (12.3)	27 (39.7)
Fatigue	10 (15.4)	57 (83.8)
Leukopenia	32 (49.2)	40 (58.8)
Thrombocytopenia	4 (6.2)	27 (39.7)
Anemia	3 (4.6)	23 (33.8)
Radiation dermatitis	21 (32.3)	/
Neurotoxicity	/	60 (88.2)
Hand foot syndrome	0	19 (27.9)
Grade		
0	9 (13.8)	0
1	36 (55.4)	18 (26.5)
2	19 (29.2)	30 (44.1)
3	1 (1.5)	17 (25)
4	0	3 (4.4)

All values are presented as number of patients followed by percentages in parentheses.

**Table 5.** Grade 3–4 adverse events in total neoadjuvant treatment

Characteristic	Total (*n *=* *21)	^a^Induction chemotherapy (*n *=* *8)	^b^Chemoradiotherapy (*n *=* *1)	^c^Consolidation chemotherapy (*n *=* *12)
Diarrhea	4 (5.9)	1 (12.5)	0	3 (25)
Vomiting	3 (4.4)	1 (12.5)	0	2 (16.7)
Fatigue	4 (5.9)	2 (25.0)	0	2 (16.7)
Leukopenia	3 (4.4)	1 (12.5)	0	2 (16.7)
Thrombocytopenia	4 (5.9)	1 (12.5)	0	3 (25.0)
Anemia	1 (1.5)	1 (12.5)	0	0
Radiation dermatitis	1 (1.5)	/	1 (1.5)	/
Neurotoxicity	1 (1.5)	1 (12.5)	/	0

All values are presented as number of patients followed by percentages in parentheses.

a A total of 68 patients received induction chemotherapy.

b A total of 65 patients received chemoradiotherapy.

c A total of 61 patients received consolidation chemotherapy.

### Surgical and pathological results

A total of 50 patients underwent TME and the sphincter preservation rate was 74.0% (37/50). The surgical and pathological results are shown in Table 6. Ten patients (20%) achieved pCR. The ypTNM Stages I, II, and III were observed in 14 (28.0%), 12 (24.0%), and 14 (28.0%) patients, respectively. Twelve (24.0%), 18 (36.0%), 20 (40.0%), and 0 (0%) patients had TRG classifications of 0, 1, 2, and 3, respectively. The post-operative 30-day morbidity and mortality rates were 12.0% and 0%, respectively, in the 50 surgical patients. The post-operative complications included obstruction, anastomotic leakage, bleeding, and pulmonary infection. Two patients underwent an unplanned reoperation (one for obstruction and the other for anastomotic leakage).

**Table 6.** Surgical and pathological results of 50 patients with rectal cancer

Characteristic	Value
Operation, *n* (%)	
LAR	24 (48.0)
APR	17 (34.0)
TaTME	7 (14.0)
TPE	1 (2.0)
Hartmann	1 (2.0)
Length of operation time, min, mean ± SD	207 ± 52
Blood loss, mL, mean ± SD	110 ± 30
Surgical complications, *n* (%)	6 (12.0)
(Clavien—Dindo classification)	
I–IIIa	4 (8.0)
IIIb–V	2 (4.0)
ypTNM, *n* (%)	
ypT0N0M0	10 (20.0)
ypT1–2N0M0	14 (28.0)
ypT3–4N0M0	12 (24.0)
ypTanyN^+^M0	14 (28.0)
TRG, *n* (%)	
0	12 (24.0)
1	18 (36.0)
2	20 (40.0)
3	0 (0.0)
CRM, *n* (%)	
Positive	1 (2.0)
Negative	49 (98.0)
LVI, *n* (%)	
Positive	4 (8.0)
Negative	46 (92.0)
PNI, *n* (%)	
Positive	9 (18.0)
Negative	41 (82.0)

LAR, low anterior resection; APR, abdominoperineal resection; TaTME, trans anal total mesorectal exsion; TPE, total pelvic excision; pCR, pathological complete response; TRG, tumor regression grade; CRM, circumferential resection margin; LVI, lymphovascular invasion; PNI, peripheral nerve invasion.

### Long-term outcomes

**Figure 4.**
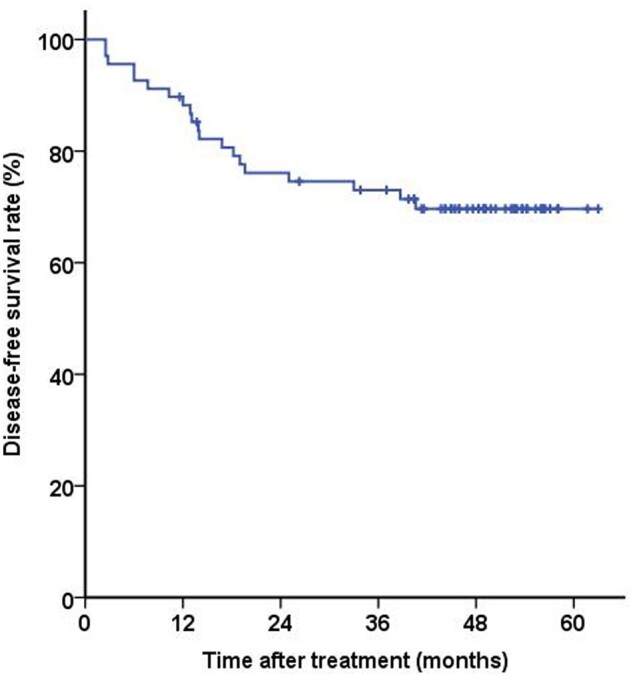
Disease-free survival of 50 patients undergoing surgical treatment.

After a median follow-up of 49.2 months (range, 39–63 months), the 3-year DFS of the entire cohort was 69.7% (Figure 4). Among the 50 patients who underwent TME, 12 experienced recurrence (two with both local and distant metastasis; 10 with distant metastasis only). The 3-year DFS of patients who underwent surgery was 79.4%. Three of the four patients who refused TME after TNT developed distant metastasis.

After a median follow-up of 45.1 months (range, 39.7–54.3 months), among the nine patients with cCR, eight still had no evidence of disease. One patient developed local regrowth at 25.8 months and received abdominoperineal resection (APR) surgery. The pathological stage was ypT3N0.

## Discussion

In this prospective Phase II study, we focused on rectal cancer patients with a high risk of distant metastasis. The TNT regimen of CRT and induction/consolidation chemotherapy had a high adverse event rate but a favorable outcome. However, distant metastasis concerns are exacerbated when the period of TNT is too long, especially for the patients with high risk factors.

Traditional neoadjuvant CRT aiming at local control has reached the upper limit of curative effect, but the OS cannot be further improved due to distant metastasis. Therefore, it is necessary to administer different treatment strategies based on the risks of recurrence. Previous studies mostly focused on patients with cT3–4 or N^+^ disease. However, the same treatment strategy seems to not be suitable for all patients, especially those with high risk factors, such as MRF^+^, EMVI^+^, or cN2 disease. For patients with rectal cancer with a high-risk high metastatic potential, more intensive preoperative treatment such as TNT seems to be a good choice [17]. Two meta-analyses showed that TNT in locally advanced rectal cancer is associated with a significant improvement in the overall pCR rate, DFS, OS, or distant metastasis-free survival compared with standard treatment [5, 18].

As in most TNT studies, we performed three cycles of induction chemotherapy before CRT. Because of the high risk of metastasis potential, induction chemotherapy is helpful to eliminate micrometastasis, evaluate the response of the tumor to chemotherapy, and reduce chemotherapy-related toxicities. Previous studies have reported that two to four cycles of oxaliplatin and capecitabine or FOLFOX chemotherapy can achieve a pCR rate of 10%–33% [18]. The CAO/ARO/AIO-12 study showed that consolidation chemotherapy led to a higher pCR rate than induction chemotherapy and a similar 3-year DFS rate [19, 20]. Although the CAO/ARO/AIO-04 study demonstrated the advantage of oxaliplatin every 2 weeks with concurrent CRT for DFS improvement, other randomized studies showed that oxaliplatin does not cause tumor shrinkage and has high toxicity [21, 22].Therefore, in this study, we did not administer oxaliplatin during CRT.

Hematological toxicity and gastrointestinal toxicity were the most common adverse events, which is consistent with the results of previous studies [23, 24]. IMRT was used in this study and the toxicity associated with radiotherapy was not high. Only one patient had Grade 3 radiation dermatitis and most adverse events were chemotherapy-related. The GCR-3 study reported a rate of Grade 3–4 adverse events from radiotherapy of 19%, which was significantly lower than that from adjuvant chemotherapy (54%) [4]. In the CAO/ARO/AIO-12 study, induction chemotherapy before CRT led to a higher rate of Grade 3–4 adverse events than consolidation chemotherapy after radiotherapy and the treatment completion rate of CRT plus consolidation chemotherapy was higher than that of induction chemotherapy before CRT [20]. However, in this study, the rate of Grade 3–4 adverse events was ∼30%, which is higher than that of most TNT regimens reported. This may be because the duration and intensity of chemotherapy in this study exceeded those of most TNT regimens reported. Notably, most Grade 3–4 adverse events occurred during consolidation chemotherapy, meaning that the tolerance to consolidation chemotherapy decreased significantly after induction chemotherapy and CRT. Therefore, although the survival benefit of adjuvant chemotherapy is unclear, consolidation chemotherapy has a higher completion rate than adjuvant chemotherapy; therefore, shifting the chemotherapy forward can significantly improve the treatment compliance. In this study, 88.2% of patients received the entire course of treatment.

All surgical patients achieved R0 resection and the positivity rate of the circumferential margin was low. The complication rate (12%) and severity of complications within 30 days after surgery were similar to those previously reported, indicating that TME is safe after TNT and TNT does not increase the risk of surgery [25]. In addition, all the patients achieved Grade 2 or better TRG classification based on the NCCN standard and nearly half of the patients had pathological Stage 0 or I disease at surgery, indicating that intensive preoperative treatment can achieve good pathological tumor regression. The 3-year DFS of all patients in the present study was 69.7%, which is lower than those of our previous study and the RAPIDO and PRODIGE 23 trials, which can be explained by the high risk factors and the four patients who refused surgery [24, 26, 27].

Organ preservation has recently become an important concern in cancer therapy in recent years. Some patients with major tumor regression after radiotherapy and chemotherapy can undergo the wait and watch strategy instead of surgery with strict evaluation [12]. The proportion of pCR and cCR in this study was 29.4%, which was close to the average pCR rate of 22% reported in previous studies and higher than the 9% in the CAO/ARO/AIO-94 study (17% when oxaliplatin was added) [23]. When cCR is observed, surgery can be avoided or delayed, and if tumor regrowth is observed during follow-up, salvage surgery can be performed without decreasing survival [28]. In a meta-analysis of 22 studies using a watch and wait strategy, 95% of patients with tumor regrowth received salvage treatment and no decrease in OS was observed [29]. In this study, only one patient had local regrowth and received surgical treatment thereafter, and other patients had a sustained response for >3 years.

Another potential disadvantage of TNT is the risk of distant metastasis during the prolonged treatment interval. Some patients who are not sensitive to CRT may have disease progression. Traditional neoadjuvant chemotherapy schedule had an interval of 6–8 weeks after conventional CRT before TME, during which the patients did not receive any treatment. We administered two cycles of consolidation chemotherapy to make full use of this period after considering the risk of tumor progression, trying to further increase the tumor response and explore the tolerance to treatment. In this study, the duration of preoperative treatment was 6 months and the patients all had at least one high risk factor. Therefore, we set up two evaluations, with the purpose of not only observing the response to treatment, but also identifying distant metastasis. We did not find this evaluation design during treatment in other similar studies. Five of our patients developed unresectable distant metastasis during TNT. All five patients had at least two high risk factors and three of them had very high CEA levels (>50 ng/mL). This indicated that PET/CT maybe need to explore potential micrometastases for such patients. In the STAR-01 trial, 3% of patients who were administered traditional preoperative CRT developed distant metastasis during the CRT and waiting interval, and 5% developed metastasis during this period in the CAO/ARO/AIO-04 trial [22, 23]. The proportion of distant metastasis in this study was higher than that in previous reports, suggesting that we should pay attention to the risk of distant metastasis when the preoperative treatment time is long, especially for patients with a high risk of distant metastasis.

Multiple modes of TNT have been assessed, including CRT combined with chemotherapy and induction chemotherapy combined with CRT and consolidation chemotherapy. The CAO/ARO/AIO-12 trial was the only randomized–controlled trial to compare different TNT regimens. Induction chemotherapy showed better chemotherapy tolerance than consolidation chemotherapy, but the pCR rate was lower [20]. In the RAPIDO trial, preoperative short-term radiotherapy and chemotherapy had a higher pCR rate and a 6.8% reduction in the 3-year distant metastasis rate compared with standard radiotherapy [24]. The PRODIGE 23 trial showed that FOLFIRINOX and CRT had a higher pCR rate, longer 3-year DFS, and better overall tolerance of chemotherapy than preoperative CRT [26]. The STELLAR trial showed that, for locally advanced rectal cancer with high risk factors, preoperative short-term radiotherapy combined with chemotherapy was not inferior to long-term radiotherapy and chemotherapy, and could be used as an alternative to CRT [30]. The positive results give confidence in the administration of TNT for Stage II–III rectal cancer. However, the huge difference in regimens between these trials challenges the universality and applicability in translating their findings into practical clinical recommendations.

There are some limitations in this study. First, it is a Phase II non-randomized study with a small sample in a single center. The sample size was determined based on the adverse event rate rather than DFS or OS. Second, some patients with potential Stage IV rectal cancer were enrolled in the study, resulting in a high metastasis rate during treatment. Third, DFS was only used as the secondary end point and was not compared with the survival rate of rectal cancer patients receiving traditional CRT treatment at the same period. Whether this regimen can be transformed into survival benefit was unclear.

Despite the limitations described above, our study showed that this treatment scheme of TNT had a high adverse event rate but favorable survival for rectal cancer patients in the Chinese population and provided a reference for how to balance adverse events and prognosis for such treatment strategies in the future.

## Authors’ Contributions

L.W., Y.C., Y.S.S., Y.H.L., W.H.W., and W.A.W. conceived and designed the project. P.J.C., T.T.S., Y.F.Y., Y.F.P., J.Z., T.C.Z., J.H.L., X.Y.Z., and Z.W.L. collected the data. P.J.C., L.W., and T.T.S. analysed and interpreted the data. P.J.C. drafted the manuscript. All authors read and approved the final manuscript.
